# Differential pattern of brain functional connectome in obsessive-compulsive disorder versus healthy controls

**DOI:** 10.17179/excli2018-1757

**Published:** 2018-11-08

**Authors:** Saeid Yazdi-Ravandi, Hassan Akhavanpour, Farshid Shamsaei, Nasrin Matinnia, Mohammad Ahmadpanah, Ali Ghaleiha, Reza Khosrowabadi

**Affiliations:** 1 Behavioral Disorders and Substance Abuse Research Center, Hamadan University of Medical Sciences, Hamadan, Iran; 2 Institute for Cognitive and Brain Sciences, Shahid Beheshti University GC, Tehran, Iran; 3 Department of Nursing, College of Basic Science, Hamadan Branch, Islamic Azad University, Hamadan, Iran

**Keywords:** electroencephalography (EEG), obsessive-compulsive disorder (OCD), functional connectivity (FC), weighted phase lag index (WPLI)

## Abstract

Researchers believe that recognition of functional impairment in some of brain networks such as frontal-parietal, default mode network (DMN), anterior medial prefrontal cortex (MPFC) and striatal structures could be a beneficial biomarker for diagnosis of obsessive-compulsive disorder (OCD). Although it is well recognized brain functional connectome in OCD patients shows changes, debate still remains on characteristics of the changes. In this regard, little has been done so far to statistically assess the altered pattern using whole brain electroencephalography. In this study, resting state EEG data of 39 outpatients with OCD and 19 healthy controls (HC) were recorded. After, brain functional network was estimated from the cleaned EEG data using the weighted phase lag index algorithm. Output matrices of OCD group and HCs were then statistically compared to represent meaningful differences. Significant differences in functional connectivity pattern were demonstrated in several regions. As expected the most significant changes were observed in frontal cortex, more significant in frontal-temporal connections (between F3 and F7, and T5 regions). These results in OCD patients are consistent with previous studies and confirm the role of frontal and temporal brain regions in OCD.

## Introduction

Obsessive-compulsive disorder (OCD) is a neuropsychiatric disorder with a lifetime occurrence of 2-3 % (Horwath and Weissman, 2000[30]). OCD is a common mental illness characterized by the obsessions and/or compulsions. Obsessions are unwanted, intrusive and recurrent thoughts that are frequently repeated and usually person attempts to relieve the distress of disturbing thoughts by repeated behaviors and/or repetitive mental acts in order to reduce or prevent anxiety and some dreaded conditions or events (compulsions) (American Psychiatric Association, 2013[5]). 

The notable symptoms of OCD are not always described by the abnormal behaviors; instead, it can also contain deficits in cognitive functions including delayed response inhibition, impaired attentional set shifting, planning, decision-making (Abramovitch et al., 2013[1]; Yazdi-Ravandi et al., 2018[68]), visual-spatial skills, and speed of information processing (Greisberg and McKay, 2003[24]; Keefe, 1995[33]; Kuelz et al., 2004[34]; Rao et al., 2008[49]; Shin et al., 2008[56]). Moreover, studies over the past two decades have shown that the brain structural and functional changes in OCD are associated with patients abnormal behavior and cognitive dysfunction (Benzina et al., 2016[11]; Friedlander and Desrocher, 2006[20]; Lewin et al., 2014[35]; Radmanesh et al., 2008[48]; Zhuo et al., 2017[70]). For instance, decreased volume of caudate nucleus (Parmar and Sarkar, 2016[45]; Robinson et al., 1995[51]), Globus pallidus, putamen and striatal region (Ferrari et al., 2008[18]) and overall cortex size have been reported in OCD patients as compared to healthy controls (Bedard et al., 2009[10]; Szeszko et al., 1999[61]). In addition, recent neurobiological studies of OCD recommend that the clinical symptoms and cognitive functions such as executive functions (Burguiere et al., 2015[13]), working memory (Li and Mody, 2016[36]), inhibitory control, (Ham et al., 2013[27]) of OCD individuals are associated with dysfunction in the cortico-striato-thalamo-cortical circuitry. The dysfunctions of cortico-striato-thalamo-cortical circuitry are mainly observed at the orbitofrontal cortex (OFC), dorsolateral prefrontal cortex, and caudate nucleus (Alexander et al., 1986[4]; Groenewegen and Uylings, 2000[25]; Rotge et al., 2009[52]; Saxena and Rauch, 2000[55]). It is supposed that this circuit is explicitly linked to the executive cognitive functions (Cavedini et al., 2006[14]). 

Alteration of frontal cortex connections to basal ganglia (Strobel et al., 2007[59]), and striatum (Melloni et al., 2012[38]; Nakao et al., 2014[40]; Arnsten and Casey, 2011[7]; Robbins et al., 2012[50]) have also been reported in the OCD which present a hyperactivity in the limbic system and the associated cortical regions (Chamberlain et al., 2008[16]). These functional changes also associate with behavioral and cognitive deficits in OCD. For instance, hyperactivity of frontal cortex is negatively correlated with neuropsychological model of the disorder (Ruchsow et al., 2007[54]) which is interpreted as a sign of disrupted inhibitory process (Adler et al., 2000[3]) and decision making (Noël et al., 2006[41]). In addition, alteration of connection between cortical regions and subcortical regions such as striatum and thalamus could also influence diverse computational activities, including reward processing, habit formation, motor control and action selection (Arnsten and Casey, 2011[7]; Robbins et al., 2012[50]) which are observed in OCD. 

Although alterations of brain functions in OCD have been investigated using various neuroimaging modalities, relatively less is known about using the electroencephalography technique (Ortigue et al., 2009[43]). Previous EEG studies have shown a decreased beta and an increased theta oscillation in OCD patients mainly at the frontal and the fronto-temporal regions (Prichep et al., 1993[46]; Locatelli et al., 1996[37]; Bucci et al., 2004[12]). In addition, changes in frontal asymmetry at lower alpha band (Wiedemann et al., 1999[66]; Ischebeck et al., 2014[31]), lower EEG complexities at the prefrontal and the fronto-temporal regions have also been reported (Aydin et al., 2015[9]). Moreover, functional communication between several brain regions entitled as functional network are also disrupted (Desarkar et al., 2007[17]; Velikova et al., 2010[63]). The functional networks could be investigated using various techniques including coherence (Velikova et al., 2010[63]), partial cross correlation (Jalili and Knyazeva, 2011[32]), mutual information (Aydin et al., 2015[9]) and phase lag index (Stam et al., 2007[58]; Vinck et al., 2011[64]). In this study, we hypothesized that alteration of functional connectivity pattern in OCD occurs in a frequency specific manner. Our aim was to investigate the potential of using this pattern as a biomarker in clinical applications. However, functional connectivity index measured by EEG signal is very sensitive to noise. Therefore, the most test-retest reliable method, weighted phase lag index (WPLI), was chosen to estimate the functional connectivity pattern. The WPLI is an extension of phase lag index that calculate a measure of phase synchrony between two signals considering a consistent lag between their phases. It has been shown that the WPLI is less sensitive to zero-lag phase-relations that could be caused by volume conduction effects (Hardmeier et al., 2014[28]; Ortiz et al., 2012[44]). Using the WPLI, the significant altered connections and the altered frequency bands were examined which are described in the following sections. 

## Materials and Methods

### Participants

The current study was conducted in psychiatric ward of the Farshchian (Sina) hospital of Hamadan city in 2016. Thirty-nine patients (25 female, age: 34.76±10.35 years) meeting DSM-IV-TR criteria for OCD and 19 healthy controls (11 female, age: 31.94 ± 8.22 years) who matched with OCD group were recruited. The study was reviewed and approved by the local ethical committee of the Hamadan University of Medical Sciences. An informed written consent was obtained from each subject prior to participation in the study. All patients were taking selective serotonin reuptake inhibitors (SSRIs). Patients with following criteria were included in this study: a) OCD diagnosis by a psychiatrist according to the DSM-IV-TR criteria and based on structured clinical interview, b) an age range between 18 and 60 years, and c) at least score of 16 on the Yale-Brown obsessive-compulsive scale (Y-BOCS). In addition, the following exclusion criteria was also considered: a) any current psychiatric disorder other than OCD diagnosed, b) history of drug and/or alcohol abuse or dependency, C) any serious concomitant general medical condition or neurologic disease, d) history of serious head injury e) intellectual disability, f) electroconvulsive therapy in the last year, g) physical disability (e.g. blindness, deaf, speech problems, paralysis and amputation), h) pregnancy and any clinical conditions that could significantly affect the EEG. In addition, healthy subject with life time and current of clinical psychiatric disorders were excluded from the study. Table 1(Tab. 1) presents some important statistical information about the two groups involved in this study. The gender, handedness and age were not significantly different between the groups, therefore, their impacts on the final outcome was ignored. Demographic and clinical information was obtained from a semi-structured interview. OCD severity was also assessed using the Yale-Brown Obsessive-Compulsive Scale (YBOCS) (Goodman et al., 1989[23]). 

### Neurophysiological data

**Figure 1:**
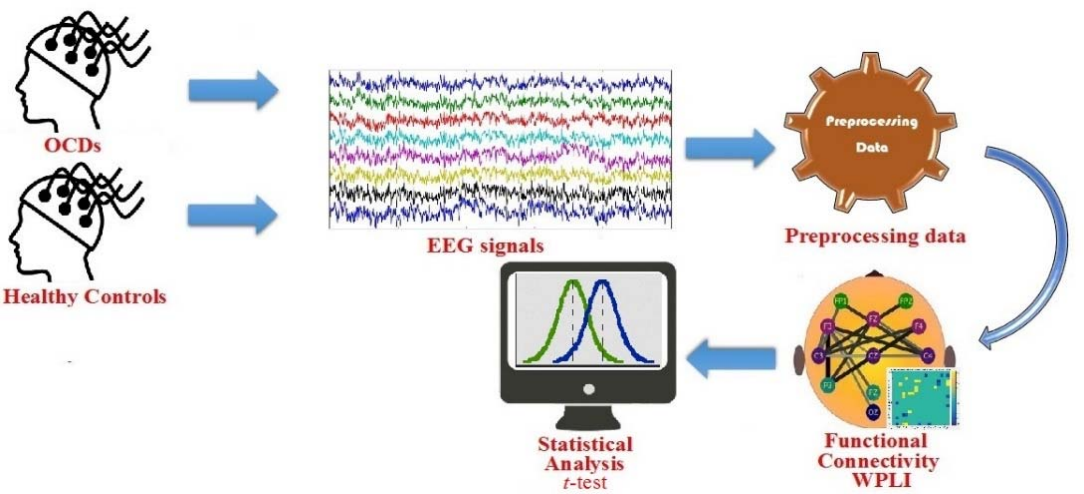
Experimental design

The EEG data acquisition was carried out between 9 and 11 a. m. on the Farshchian Sina hospital of Hamadan city using a Cadwell Easy II Amplifier with 19 Ag/AgCl surface electrodes including FP1, F3, F7, C3, T3, T5, P3, O1, Fz, Cz, Pz, FP2, F4, F8, C4, T4, T6, P4 and O2. The electrodes were placed on the scalp according to the 10-20 international system via Electro-Caps (Electro-Cap International, Inc.) with Cz as the reference electrode. The electrode impedance was smaller than 5 kΩ throughout the session. The EEG data was recorded in eyes-open resting state with a sampling rate of 200 Hz. The experimental design is shown in Figure 1(Fig. 1). 

The acquired EEG signals were then band-pass filtered between 1-40 Hz using a basic FIR filter with a zero phase shift. Then, the filtered data was segmented to trials of 3 seconds. Subsequently, artifacts were removed using the independent component analysis followed by a visual inspection. The channels diagnosed as bad using the kurtosis measure were interpolated. Then, the re-referenced EEG data to average channels were used to estimate the brain functional connectivity network. Since the brain works in a frequency specific manner, the functional connectivity were calculated for each of the conventional frequency bands separately, the implied frequency bands including delta (1-4 Hz), theta (4-8 Hz), alpha I (8-10 Hz), alpha II (10-12 Hz), beta I (12-15 Hz), beta II (15-18 Hz), beta III (18-25 Hz), beta IV (25-30 Hz) and lower gamma band (30-40 Hz). The process of EEG data was entirely performed in MATLAB R2016a (The MathWorks Inc., Natrick, USA) using the EEGLAB v13.6.5b toolbox.

The functional connectivity could be estimated using various approaches such as coherence (Nunez et al., 1997[42]; Srinivasan et al., 2007[57]), partial correlation (Zhou et al., 2009[69]; Jalili and Knyazeva, 2011[32]; Wang et al., 2016[65]), mutual information (Aydin et al., 2015[9]) and phase lag index (Stam et al., 2007[58]; Vinck et al., 2011[64]). In this study, the WPLI was implied to identify interdependencies and interaction between time series of each pairs of electrodes. The WPLI has better test-retest reliability as compared to other functional connectivity measures (Hardmeier et al., 2014[28]).

### Weighted Phase Lag Index

In the first stage, Fourier transform of two real-valued signals x(t) and y(t) are computed and labeled as X(f) and Y(f). Then, X and Y are used to compute the complex cross-spectrum

C(f) = X(f)Y*(f) of two signals, 

where Y* represents the complex conjugate of Y. Subsequently, the complex non-diagonal part of C is considered as Z to focus on a particular frequency of interest f. 

Then, PLI is calculated by taking absolute value of the sign of the imaginary part of Z: PLI ≡ | E[sign(Im(Z))] |. 

In fact, uncorrelated noise sources will motive an increase of signal power and weighting of cross spectrum limits the influence of cross-spectrum elements around the real axes. The real axes have a higher probability of risk in changing their “true” sign with small noise disorderliness. Therefore, the WPLI that was proposed by Martin Vinck and colleagues (2011[64]) is calculated by weighting the cross-spectrum according to the magnitude of the imaginary component and would be more robustness to noise as compared to the PLI, coherence, and imaginary coherence (Ortiz et al., 2012[44]).

### Statistical analysis

In the final stage, group differences in functional connectivity between OCD patients and HCs were statistically evaluated. Statistical comparisons were performed using a non-parametric permutation t-test. The normality assumption for the functional connectivity was checked using the Kolmogorov-Smirnov test. Then, a two-tailed paired t-test was applied to identify the significant changes of functional connectivity in OCDs as compared to HCs. A threshold of p<0.05 (Fisher permutation) was considered and the results were corrected for the multiple comparisons error using the Bonferroni method. 

## Results

**Figure 2:**
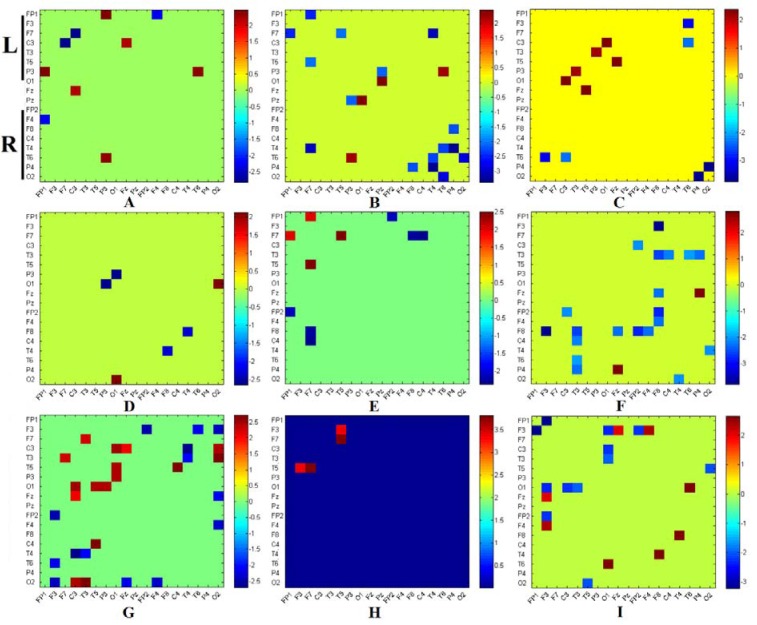
Functional connectivity differences between OCD group versus healthy individuals in various frequency band including A. Delta (1-4 Hz), B. Theta (4-8 Hz), C. Alpha I (8-10 Hz), D. Alpha II (10-12 Hz), E. Beta I (12-15 Hz), F. Beta II (15-18 Hz), G. Beta III (18-25 Hz), H. Beta IV (25-30 Hz) and I. Gamma (30-40 Hz). The color bars present the range of T value changes in each comparison. The hot colors indicate that the average WPLI of OCD group is higher than healthy controls, and the cold colors present a lower average of WPLI measures in the OCD group. The results have been masked with binary mask of their related p values and elements of the mask matrix with p values bigger than 0.05 have been marked as zero and p values lower than 0.05 have been marked as one.

Subsequently, the brain functional connectivity changes in OCDs as compared to HCs were statistically identified in 9 frequency bands. Figure 2(Fig. 2) and Table 2(Tab. 2) present the most significant differences in functional connectivity between OCD and HC groups.

The functional connectivity matrices presented in Figure 2(Fig. 2) have been rearranged to left and right part based on their channel locations at the left or right hemisphere relative to the central line. The results clearly present significant changes in functional connectivity between the frontal regions and other areas of the brain. Interestingly, these results were observed in a similar manner in all frequency bands (Table 2(Tab. 2)).

Based on the above mentioned results (Figure 2(Fig. 2), Table 2(Tab. 2)), it is obvious that connections between frontal areas and other brain regions, mainly temporal and occipital regions, are significantly altered in OCD patients. These alterations are frequency specific and mainly show decrement in synchronous activations of frontal, temporal and occipital regions. Nevertheless, increase of synchrony activities are also observed between oscillatory activations of the left frontal and the left temporal regions at the alpha and the beta bands. On the other hand, the most significant changes are observed at the frontal-temporal connections in very high beta band (Figure 2-H(Fig. 2)).

## Discussion

In this study significant differences were observed at several connections; mainly at the connections between frontal and temporal regions (F3-T5 and F7-T5). These results in OCD patients are consistent with previous studies and confirm the role of frontal and temporal brain regions in the obsessive compulsive disorder. In addition, comparison of functional connectome in OCDs and HCs was performed in a frequency-band specific manner. Nine frequency bands explained in the results section were included. Interestingly, significant changes were observed in all the frequency bands. The altered functional connectivity was distributed widely in the whole brain network. The most significant changes were observed at the very high Beta frequency band. These results demonstrate that OCD influences the whole brain network and is not locally constrained. Nevertheless, contribution of the fronto-temporal network at the very high Beta frequencies seems more relevant to OCD. 

The severity of functional connectivity changes between frontal and temporal regions at very high beta band denotes that there should be an association between the OCD symptoms and abnormalities in the mentioned regions. Notably, we also observed an increased power spectrum of EEG at the frontal region at the same frequency in the OCD group as compared to HCs. The high significance level of the results highlights the potential of using neuromarkers beside the behavioral markers to understand the OCD better and achieve to a specific and personalized treatment subsequently. Therefore, we will discuss the association between the altered behavioral and cognitive processes in the OCD and the related neural findings in the following.

The hyper-connectivity observed at the fronto-temporal connection represents reinforcement of information processing at the frontal and temporal regions. The frontal region is known to be involved in various high-level cognitive functions such as attention (Miller and Cummings, 2007[39]). Therefore, when involvement of the frontal region in process of information is increased potentially the functional connection between this region and other parts of the brain is also enhanced. For instance, an exaggerated attention to the irrelevant cues could increase the involvement of the frontal region and subsequently its functional connection to other regions. This exaggerated attention or miscarriage to filter out unimportant information is also observed in OCD individuals (Antony and Stein, 2009[6]). Dysfunction of frontal cortex could impair the inhibitory mechanism as well (Garcia-Junco-Clemente et al., 2017[21]) which is a major sign of OCD (Chamberlain et al., 2005[15]). In addition to inhibitory mechanism, the decision making process is also disrupted in OCD (Abramovitch et al., 2015[2]; Aydin et al., 2014[8]) which is also related to activity of frontal region and striatum. This incensement is mainly observed at the left frontal region and supposed to be asymmetric (Grützmann et al., 2017[26]). Although, findings on the disruptions of the frontal cortex in OCD patients are divergent (Lewin et al., 2014[35]; Nakao et al., 2014[40]; Wong et al., 2015[67]; Van den Heuvel et al., 2005[62]; Gonçalves et al., 2016[22]; Swinson et al., 2001[60]); a moderate increase of the beta band has been reported in other studies as well (Purcell et al., 1998[47]; Rubia et al., 2011[53]). The enhanced connections are observed at the frontal, temporal, parietal regions. The enhanced functional connectivity between frontal and temporal regions has been related to an excessive attention to inconsequential information (Fornito et al., 2013[19]) which is an indicator of people with ultra-high-risk for psychosis, and the auditory/verbal hallucination in schizophrenic patients (Hoffman et al., 2011[29]). Therefore, we think that exaggerated process of information at the frontal and temporal regions will increase their interconnection as well as their connectivity to other parts of the brain such as basal ganglia and cingulum in OCD patients. Since, the EEG data mostly captures the cortical activities, therefore, the most significant results were observed at the fronto-temporal connections. We hope these findings could improve theoretical construct about the influence of OCD on the brain structure and functions and open an avenue to intervention paradigms. 

## Limitation

Considering the ethical issues, we only recruited the OCD patients under medication in our study. However, the medication could influence the functionality of the brain. Therefore, the results may not be directly extended to all OCD patients.

## Notes

Ali Ghaleiha and Reza Khosrowabadi (Institute for Cognitive and Brain Sciences, Shahid Beheshti University, Evin Sq., Tehran 19839-63113, Iran; Tel: +98(0)9101738501, E-mail: r_khosroabadi@sbu.ac.ir) contributed equally as corresponding authors.

## Acknowledgement

This article is part of a PhD thesis supported by Hamadan University of Medical Sciences (Grant No: 940125303). The authors gratefully acknowledge the financial support provided by vice chancellor of research and technology of Hamadan University of Medical Sciences.

## Figures and Tables

**Table 1:**
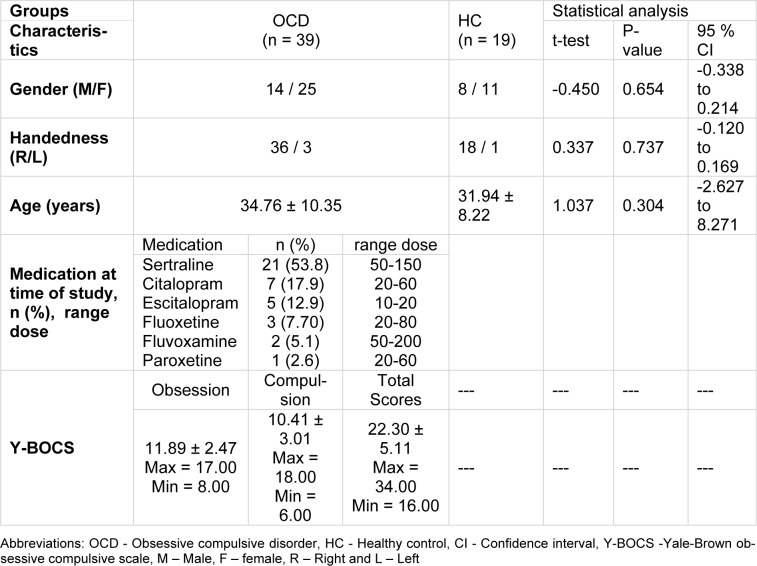
Demographic characteristics of the participants in this study

**Table 2:**
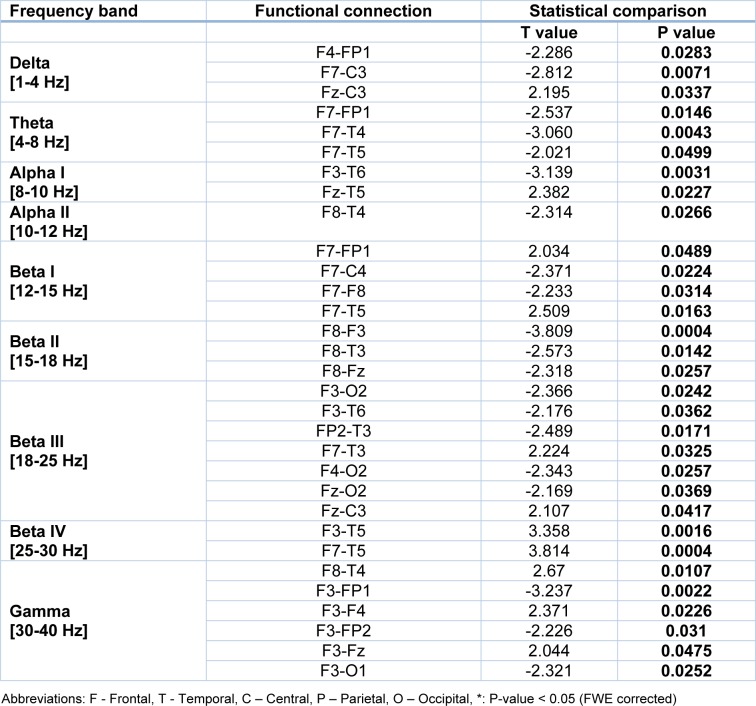
Significant changes in the brain functional network of the OCD individuals as compared to healthy controls
